# Factors Affecting Health-Promoting Behaviors in Patients with Cardiovascular Disease

**DOI:** 10.3390/healthcare9010060

**Published:** 2021-01-08

**Authors:** Hwan-Cheol Park, Jihyun Oh

**Affiliations:** 1 Division of Cardiology, Department of Internal Medicine, Hanyang University Guri Hospital, Guri City 11923, Korea; hjpooh74@hanyang.ac.kr; 2 Department of Nursing, Daejeon University, Daejeon 34520, Korea

**Keywords:** cardiovascular disease, health behavior, physical activity, depression, anxiety

## Abstract

Cardiovascular disease is the leading cause of death globally and the second most common cause of death in South Korea. Health-promoting behaviors recommended for patients with cardiovascular disease include control of diet, physical activity, cessation of smoking, medication adherence, and adherence to medical recommendations. This study aimed to determine the relationship between depression, anxiety, perception of health status, and health-promoting behavior in patients from South Korea who have suffered from cardiovascular disease. The study population comprised 161 patients at the cardiovascular center at H Hospital who were diagnosed with cardiovascular disease. Descriptive statistics and stepwise multiple regression were employed to analyze the data. Negative correlations existed between depression, perception of health status, and health-promoting behavior. By contrast, a positive correlation existed between the perception of health status and health-promoting behavior. The main factors affecting health-promoting behaviors were alcohol consumption, duration of diagnosis, perception of health status, and depression. These variables explained 15.8% of the variance. To prevent adverse cardiac events, patients who suffer from cardiovascular disease should be assessed as soon as possible to identify psychiatric symptoms, thereby developing a potential intervention aimed at decreasing negative illness consequences.

## 1. Introduction

Cardiovascular disease (CVD), which includes heart attack and stroke, is the leading cause of death globally and the second most common cause of death in South Korea [1]. The estimated prevalence of CVD in South Korea in 2018 was 62,947 [2]. The number of deaths due to CVD was 62.4 out of every 100,000 Koreans, accounting for 10.6% of all deaths in South Korea [2].

People diagnosed with CVD may experience anxiety, depressive symptoms, and post-traumatic stress disorder [3,4]. Depression and anxiety are especially strongly associated with CVD [5,6]. These comorbidities include common mental health problems that appear to persist for months to years, adversely impacting the quality of life and interfering with such patients’ self-care behaviors [7,8,9]. Depression and anxiety among CVD patients might substantially delay or prevent such patients from eschewing unhealthy behaviors including smoking, heavy alcohol consumption, physical inactivity, and unhealthy diet. Thus, to prevent recurrence of and complications owing to CVD, patients with CVD should change their engagement in unhealthy behaviors and sustain healthy behaviors [1].

Depression is also associated with non-adherence to medication and healthcare provider recommendations, and poor adherence behavior to cardiac rehabilitation, which may result in a worse long-term prognosis, problems during recovery, or death [3,10,11]. Anxiety is a body’s stress response, which, if persistent or generalized, may contribute to cardiac attack; however, if anxiety prompts an individual to engage more in treatment (e.g., nonsmoking status, healthy diet, exercising regularly, adhering to medications, and health care utilization) [12,13] it may be beneficial [14]. On the other hand, it might be more likely to attribute anxiety to medication non-adherence [15]. Anxiety and depression are prevalent in patients with CVD [16,17]. However, a previous study did not find a statistically significant correlation between anxiety and CVD. Thus, the present study explored whether anxiety appears in patients with CVD and whether anxiety affects health-promoting behavior.

Health-promoting behaviors that have been recommended for patients with CVD include control of diet, physical activity, cessation of smoking, medication adherence, and adherence to medical recommendations (including monitoring blood pressure and body weight daily). To date, studies on health-promoting behavior in patients with CVD have focused on the relationship between depression and health behavior [18,19], patients’ symptom recognition [20], or psychiatric symptoms [1,7,21]. However, the extent to which many patients with CVD undertake health-related behaviors, how many patients with CVD suffer from psychiatric symptoms, and how psychiatric symptoms affect patients’ health-related behaviors have not been elucidated.

Individual perception of health status is the primary factor influencing the decision to improve a healthy lifestyle [22]. As an indicator of health in general, health perception is a significant predictor of health outcomes, such as quality of life and health behaviors, affecting disease prognosis and health status [23]. Nonsmoking status, regular physical activity, healthy diet, adherence to medication and medical recommendations, and psychological well-being are associated with higher perceived health status [24,25,26,27]. However, the results of studies exploring the interaction between a healthy lifestyle and subjective health perception are not consistent [28]. Therefore, the present study aimed to determine the relationship between perception of health status, anxiety, depression, and health-promoting behaviors and to identify factors affecting health-promoting behaviors among patients with CVD.

## 2. Materials and Methods

### 2.1. Study Design and Sample

This study was a cross-sectional study that investigated the relationships between perception of health status, anxiety, depression, and health-promoting behaviors in patients with CVD and explored which factors influence patients’ health-promoting behavior. The sample consisted of 161 patients diagnosed with CVD who met the following inclusion criteria: (1) at least 18 years of age; (2) able to read and comprehend the questionnaire and provide informed consent; (3) had received a diagnosis of cardiovascular disease, including myocardial infarction, angina pectoris, hypertension, stroke, and heart failure, from an attending by a cardiologist; and (4) at least 12 months between confirmation of that diagnosis and participation in this study. Only those patients who met the above criteria and agreed to participate in the study after being informed of the study purposes and procedures were included in the study. 

The patients were from H hospital in Daejeon, South Korea, who were recruited between 30 May and 23 June in 2019. For multiple regression, the minimum sample size was estimated to be 138 for a significance level of <0.05, a medium effect size of 0.15, and a power of 0.95 using G*Power 3.1 analysis software [29]. A total of 180 patients were eligible for this study. Three patients declined to participate, and sixteen patients withdrew. Thus, a total of 161 patients were included in the final analysis (response rate of 89.4%). Therefore, the sample size in this study was adequate.

### 2.2. Measurement

#### 2.2.1. Depression

Depressive symptoms were measured using the Center for Epidemiologic Studies Depression Scale (CESD), which contains 20 items for measuring depressive symptomatology in the general population [30]. Each item has response options ranging from 0 (rarely or none of the time [<1 day]) to 3 (most or all of the time [5–7 days]). Scores are calculated by summing all response options; total scores range from 0 to 60. A score of 16 points or more is considered as an indication of depression. Cronbach’s alpha (α) for this scale was 0.92, indicating high reliability.

#### 2.2.2. Anxiety

Anxiety has most frequently been assessed with the State-Trait Anxiety Inventory (STAI) developed by Spielberger, Gorsuch, and Lushene [31]. The STAI measures 2 different types of anxiety: state anxiety and trait anxiety. The State Anxiety Scale asks a patient to describe their feelings in certain conditions. A focus on state anxiety was most appropriate in this study because state anxiety will fluctuate over time as a function of individual stress and of that individual’s interpretation of a stressful situation as dangerous or threatening [31]. The State Anxiety Scale consists of a 20-item self-report questionnaire asking questions about how the respondent feels during the administration of the survey (e.g., “I feel calm”; “I feel satisfied”), which are responded to on a 4-point Likert scale with items emphasizing the intensity of particular symptoms (ranging from 1 = “not at all” to 4 = “very much so”). The total score is the sum of the item responses and can range from 20 to 80. Higher scores indicate higher levels of state anxiety. Cronbach’s α for this scale was 0.82, indicating high reliability.

#### 2.2.3. Perception of Health Status

Perception of health status was assessed by a single item asking, “How is your overall health at the present time?” [32]. Items were responded to using a 5-point Likert scale ranging from 1 (“very poor”) to 5 (“very good”). Higher scores indicate higher perceived health status.

#### 2.2.4. Health-Promoting Behaviors

Song [33] developed a measure to assess an individual’s level of health-promoting behavior. There are 30 items that are responded to using a 4-point Likert scale (1 = “never” to 4 = “often”) and that cover 5 dimensions of health behaviors: nutrition status, physical activity, smoking habit, medication adherence, and adherence to medical recommendations. The higher a respondent’s score, the higher that respondent’s level of health-promoting behavior. Cronbach’s α for this study was 0.84, indicating high reliability.

### 2.3. Data Analysis

The data were statistically analyzed using SPSS Statistics 22.0 software (IBM Corp., Armonk, NY, USA). Participants’ general characteristics were analyzed with frequencies and percentages, and level of depression, anxiety, perception of health status, and health-promoting behavior were analyzed by calculating the mean and standard deviation. Differences in health-promoting behavior concerning general characteristics were analyzed using a *t*-test and an analysis of variance [34]. The *t*-test was performed assuming that the figures are normally distributed. Scheffe’s test was used as a post-hoc test in the analysis of variance. The correlations between health-promoting behavior and related factors were analyzed using Pearson’s correlation coefficients. A stepwise multiple regression was used to examine the factors influencing health-promoting behavior.

### 2.4. Ethical Considerations

Research ethics approval for the present study was received by the Research Committee of Daejeon University (IRB no. 1040647-201904-HR-014-02). Questionnaires were directly distributed only to patients who signed a written consent form after being told about the following issues: the study aims, data collection process, benefits and risks of participation, ability to withdraw from the study at any time, protection of personal information and confidentiality, and the use of data only for research purposes. Patients completed the questionnaire in the presence of the researcher and returned it upon completion. Completing the questionnaire took approximately 20 min.

## 3. Results

### 3.1. Participants’ General Characteristics and Differences among the Participants

Participants’ characteristics are described in Table 1. One hundred sixty-one patients out of 180 fully participated in the study (response rate of 89.4%), ranging from 29 to 87 years of age (*M* = 66.3, *SD* = 10.7). Half of the participants were male (50.3%). A large majority of the participants reported being married (86.3%), and the proportion of those who received an education beyond the undergraduate level (35.4%) and of those who received a high school education (30.4%) were similar. More than half of the participants (69.6%) reported their economic status to be higher than the middle level. Slightly more than half had one comorbid condition (52.8%) and did not consume any alcohol (59.0%). The mean duration since diagnosis of CVD was 6.9 years and ranged from 1 to 21 years. The greatest number of participants reported being diagnosed 1 to 10 years ago (68.3%). Scores on the CESD indicated that 85 participants (51.6%) were depressed.

There were differences in health-promoting behaviors according to education level (*F* = 5.470, *p* = 0.001), alcohol consumption (*t* = 3.093, *p* = 0.002), duration of diagnosis (*t* = −2.197, *p* = 0.029), and depression status (*t* = −2.831, *p* = 0.005). Women practiced significantly better health-promoting behaviors (*t* = −2.395, *p* = 0.018) than men. Post hoc testing showed that participants who graduated from high school had a mean score that was significantly lower than those who had completed an undergraduate education or beyond.

In the subdomains of health-promoting behaviors, with regard to adherence to medication there was a significant difference based on economic status (*t* = 2.317, *p* = 0.022) and depression status (*t* = −2.758, *p* = 0.006). Regarding differences in adherence to dietary habits, there were significant differences according to alcohol consumption (*t* = 3.189, *p* = 0.002), duration from diagnosis of CVD (*t* = −2.566, *p* = 0.011), and depression status (*t* = −2.300, *p* = 0.023). Further, women practiced significantly better adherence to dietary habits (*t* = −3.319, *p* = 0.001) than men. There were differences in adherence to physical activity according to marital status (*t* = 2.390, *p* = 0.022), education level (*F* = 8.009, *p* < 0.001), alcohol consumption (*t* = 2.301, *p* = 0.023), and duration from diagnosis of CVD (*t* = −3.032, *p* = 0.003). Post hoc testing showed that adherence to physical activity was the lowest in participants who had a high school education compared to those with no education or an elementary school, middle school, or undergraduate school education. There were differences in adherence to medical recommendations according to participants’ education level (*F* = 5.699, *p* = 0.001), economic status (*t* = 3.932, *p* < 0.001), alcohol consumption (*t* = 3.541, *p* = 0.001), and depression status (*t* = −3.456, *p* = 0.001). Post hoc testing showed that participants who had graduated middle school had a significantly higher mean score compared to those who had not graduated elementary school, had graduated elementary school, or had graduated high school. Regarding adherence to nonsmoking, there were significant differences based on marital status (*t* = 4.190, *p* < 0.001) and economic status (*t* = 2.366, *p* = 0.019).

### 3.2. Perception of Health Status, Depression, Anxiety, and Health-Promoting Behaviors

Levels of depression, anxiety, perception of health status, and health-promoting behaviors are presented in Table 2. The mean score for depression was 21.72 (*SD* = 10.00), for anxiety was 2.15 (*SD* = 0.37), and for perception of health status was 2.61 (*SD* = 1.01). The total mean score for health-promoting behavior was 2.66 (*SD* = 0.41). Among the five dimensions of health-promoting behavior, the highest score was for quitting smoking and the lowest score was for physical activity.

### 3.3. Correlations between Perception of Health Status, Depression, Anxiety, and Health-Promoting Behaviors

Correlations among the variables are presented in Table 3. Depression showed significant negative correlations with perception of health status (*r* = −0.428, *p* < 0.001) and health-promoting behavior (*r* = −0.323, *p* = 0.002). Perception of health status showed a significant positive correlation with health-promoting behavior (*r* = 0.203, *p =* 0.010). These results indicate that the higher the level of depression, the less one engaged in health-promoting behavior, and the more favorable one’s perception of their health status is, the more likely the individual also engaged in health-promoting behavior.

### 3.4. Factors Influencing Health-Promoting Behaviors

Factors influencing health-promoting behaviors are shown in Table 4. Perception of health status, depression, anxiety, and particular general characteristics differed in how they influenced health-promoting behavior in patients with CVD, as shown in the regression models. The general characteristics were gender, marital status, education level, economic status, alcohol consumption, and duration of diagnosis. Using stepwise multiple regression analysis to identify the factors that affect health-promoting behaviors, duration of diagnosis (β = 0.245, *p* = 0.001), depression (β = 0.232, *p* = 0.003), alcohol consumption (β = −0.204, *p* = 0.006), and perception of health status (β = 0.183, *p* = 0.014) were significant factors. These variables explained 15.8% of the variance in health-promoting behavior (*F* = 8.487, *p* < 0.001, *R*^2^ = 0.1979, Adj. *R*^2^ = 0.158).

In the subdomains of health-promoting behavior, the factors influencing medication adherence were perception of health status (β = 0.362, *p* < 0.001) and depression (β = 0.165, *p* = 0.026). The explanatory power of these variables was 16.4% (*F* = 16.639, *p* < 0.001, *R*^2^ = 0.174, Adj. *R*^2^ = 0.164). The factors influencing dietary adherence were duration of diagnosis (β = 0.230, *p =* 0.004), depression (β = 0.213, *p =* 0.007), and gender (β = 0.198, *p* = 0.010). The explanatory power of these variables was 11.8% (*F* = 8.146, *p* < 0.001, *R*^2^ = 0.135, Adj. *R*^2^ = 0.118). The factors influencing adherence to physical activity were duration from diagnosis of CVD (β = 0.248, *p* = 0.001), perception of health status (β = 0.200, *p* = 0.008), and alcohol consumption (β = −0.174, *p* = 0.011). The explanatory power of these variables was 11.0% (*F* = 7.607, *p* < 0.01, *R*^2^ = 0.127, Adj. *R*^2^ = 0.110). The factors influencing adherence to medical recommendation were economic status (β = −0.301, *p* < 0.001), alcohol consumption (β = −0.251, *p* = 0.001), and depression (β = 0.227, *p* = 0.002). The explanatory power of these variables was 20.2% (*F* = 14.529, *p* < 0.001, *R*^2^ = 0.217, Adj. *R*^2^ = 0.202). The factors influencing adherence to nonsmoking were perception of health status (β = 0.293, *p* < 0.001), anxiety (β = −0.262, *p* = 0.001), marital status (β = −0.233, *p* = 0.003), and economic status (β = −0.147, *p* = 0.048). The explanatory power of these variables was 17.3% (*F* = 9.395, *p* < 0.001, *R*^2^ = 0.194, Adj. *R*^2^ = 0.173).

## 4. Discussion

The present study aimed to determine the relationship between depression, anxiety, perception of health status, and health-promoting behaviors in patients diagnosed with cardiovascular disease and to identify factors affecting health-promoting behaviors.

The mean score for the scale assessing health-promoting behavior was 2.66, which was higher than the median value. The mean score for health-promoting behaviors in adolescents with cardiogenic heart disease [35] was considerably higher than the mean in our sample. Moreover, the mean score for health-promoting behavior in our participants was slightly higher than that found in a sample of older adults with osteoarthritis [36]. This finding suggests that with the progression of CVD, health-promoting interventions should consider age, different symptoms according to current health status, and duration of diagnosis of cardiovascular disease. Moreover, women engaged in health-promoting behavior more than men. Of the five dimensions of health-promoting behaviors in this study, adherence to nonsmoking and physical activity received the highest and the lowest mean scores, respectively.

The present study’s findings indicate that physical activity received the lowest score among health-promoting behaviors. Adherence to physical activity, as the first treatment in primary prevention, decreases mortality owing to CVD. During long-term follow-up, focusing and increasing the intensity of physical activity have together served as a significant predictor of survival after myocardial infarction [37] and reduced risk of lifestyle-related diseases such as stroke and CVD [38]. Although the physical activity levels of the participants had the lowest score among the health-promoting behaviors in this study, there is evidence to suggest that health programs to increase the effects of physical activity should be applied to patients with CVD. Physical activity is still underutilized as a component of preventive and treatment strategies in health care [37]; thus, maintaining or increasing patients’ level of physical activity reinforces the importance of a healthy lifestyle and highlights the need for preventing complications of CVD and lowering the risk of recurrence of CVD. Thus, higher levels of physical activity lower the risk for adverse cardiovascular events; also, engaging in physical activity or exercise programs improves mood and reduces symptoms of depression and anxiety [21]. Research indicates that the relationship between depression or anxiety and physical inactivity are well-established [7,9,39], although the findings in our study did not provide support for anxiety or depression as being an influencing factor in adherence to physical activity. Nevertheless, healthcare providers should help motivate patients to engage in regular physical activity using the body’s large muscle groups (e.g., walking, cycling, swimming). These physical activities can improve symptoms, functional capacity, and mental health while reducing the risk of CVD.

This study found that the main factors affecting adherence to medication, which was the second-highest mean score among the subdomains of health-promoting behaviors, were perception of health status and depression. Our findings are consistent with previous studies showing that depression can contribute to medication nonadherence in CVD patients [10,40,41], and the results of a study in which higher perceived health status positively correlated with medication adherence in ischemic stroke patients [42]. We found that medication adherence for those whose scores indicated they were not depressed was higher than the scores of those who were classified as depressed. This finding applied to diet adherence and medical recommendation adherence as well. These findings are consistent with previous studies on medication adherence [18,19,43]. The present study’s findings are evidence that depression is associated with cardiovascular disease risk factors including insufficient physical activity, poor adherence to medication, poor adherence to medical appointments, and insufficient diet habits. Depression significantly affects an individual’s functioning capacity and disrupts the individual’s daily functioning [43]. Our findings indicated that the mean score for depression was 21.72, which is higher than the cutoff score of 16. Considering these results, evidence from the current study suggests that a strong link between depression and CVE may be a predictor of a worse prognosis and increased mortality, and such a prognosis subsequently leads to behavioral factors including poor adherence to lifestyle modifications, lack of exercise, and medication nonadherence [44,45]. Indeed, low medication adherence has been associated with poor chronic disease control and may lead to worsening of the disease and delay the process of disease progress and treatment response [41,46,47].

In the subdomains of health-promoting behaviors, the main factors affecting medical recommendation adherence were marital status, economic status, and depression. An understanding of these associations is especially important in CVD because patients suffering from depression are less likely to adhere to dietary, medical, and medication recommendations, and poor adherence to health-promoting behaviors can lead to cardiovascular events [9,10,44].

The main factors affecting adherence to dietary habits were gender, duration of diagnosis, and depression. Women adhered more strictly to good dietary habits compared to men. This finding is consistent with those of previous studies [48,49], which revealed that women had better adherence to health-promoting behaviors than men. Traditionally, shopping for ingredients and preparing meals is a domestic role for women [50], so women are more responsible for cooking and preparing meals. This may be why women can adhere to dietary habits for a healthy diet and lifestyle to prevent CVD. The mean duration since diagnosis of CVD was 6.9 years in the present study, and the range was up to 21 years. Adherence to a diet long-term may be difficult for patients with CVD, and depression may also make it difficult to adhere to dietary constraints. These findings are important to consider given the results of a previous study indicating that a healthy diet including a high intake of fruits, nuts, and vegetables, and low-fat dairy products, and low intake of sodium correlated with lower CVD risk [51].

Concerning adherence to nonsmoking as a health-promoting behavior, marital status, economic status, perception of health status, and anxiety were identified as factors affecting adherence. All cardiac patients who have had a major cardiac event or have a history of CVD are required to abstain from smoking. Continued smoking is a significant predictor of complications and a powerful predictor of future morbidity and mortality [52]. The current findings are consistent with those of a previous study in which higher anxiety was associated with lower adherence to smoking cessation [53]. Moreover, anxiety could impair the desire and ability of the patient to follow treatment recommendations, and heavy smoking increases the risk of developing anxiety [54]. Those who experience high anxiety perceive quitting smoking as more difficult. Therefore, some smokers continue smoking to avoid experiencing feelings of stress and anxiety, although most smokers want to stop [55]. Thus, the vicious circle concerning smoking and anxiety in CVD patients repeats itself.

Our findings bring attention to those unhealthy behaviors such as smoking, poor dietary habits, alcohol consumption, and physical inactivity that should be addressed in patients with CVD through overall lifestyle interventions in the long-term to reduce cardiovascular mortality. These healthy lifestyle interventions are quite effective in reducing the risk of cardiovascular disease while lowering rehospitalization rates [56]. In addition, in the present study, half of the patients (51.6%) were depressed. The finding that depression negatively correlated with perception of health status and health-promoting behavior is in line with the findings of previous studies reporting that the onset of depressive symptoms was associated with decreased self-care behavior and lower perceived health status [48,57]. Moreover, depression may lead to a reduced healthy lifestyle, inducing a lack of regular exercise, healthy diet, medication adherence, and motivation to quit smoking. A previous study reported that drinking alcohol is linked to anxiety, but not depression [58]. Another study reported that depression is an important factor in the relationship between anxiety and alcohol dependence [59]. Although this study did not establish a link between alcohol, depression, and anxiety, future studies could explore this in greater detail. This study’s findings highlight the importance and necessity of managing depression in patients with cardiovascular disease. Indeed, the association is almost certainly bidirectional, because depression causes physical inactivity, and physical inactivity exacerbates depression. This study evaluated depression and health-promoting behavior at the same point in time; thus, whether physical inactivity was the cause or result of depression could not be determined. An understanding of these associations is especially important in treating CVD because depression has been specifically linked to increased CVD susceptibility and adverse outcomes, as well as to accelerated related readmissions and mortality. Despite the substantial body of evidence demonstrating a strong link between depression and CVD, there is an overall lack of studies on effective treatments and interventions targeting depression following a diagnosis of CVD.

### 4.1. Implication for Nursing Practice

The present study indicated that depression, anxiety, and perception of health status are significant factors associated with health-promoting behaviors in patients with CVD. To prevent adverse cardiac events, patients with CVD should be assessed early on to identify the presence of any psychiatric symptoms such as anxiety and depression after cardiovascular events. In clinical practice, these results emphasize that nurses and nurse managers can help patients to make their own decisions about health aimed at decreasing negative consequences from illness, thereby strengthening health-promoting behaviors. Structured nursing interventions for improving health-promoting behaviors are needed. Considering previous nursing intervention studies [60,61,62], patients’ continuous disease management would require combined interventions including web-based or mobile-based interventions, not just face-to-face nursing education and nursing interventions. Electronic health (eHealth) or mobile health (mHealth) interventions can induce positive behavior changes and promote healthy lifestyles [63], allowing easy access. Moreover, effective nursing strategies including medication education, post-discharge programs, and nurse provider healthcare services for CVD patients are necessary to increase health-promoting behaviors.

### 4.2. Limitations

Several limitations of this study deserve mention. First, the current study is limited in the sample’s degree of representation and generalizability. Second, the influences on the respondents’ health-promoting behavior were investigated using only three variables. Therefore, it is difficult to generalize the study results to the entire population. Third, there was no intervention to test the relationships found. Therefore, no evidence could be obtained to support the contention that reducing depression and anxiety can improve health-promoting behaviors in patients with CVD. Because this study was a descriptive quantitative study, there is the possibility of unmeasured confounding variables, and cause and effect cannot be determined.

## 5. Conclusions

This study was conducted to explore the factors affecting health-promoting behaviors in patients with CVD. Of the five dimensions of health-promoting behaviors, adherence to nonsmoking and physical activity had the highest and the lowest mean scores, respectively. Depression, anxiety, and perception of health status were each associated with health-promoting behaviors. The results also showed that gender, marital status, economic status, alcohol consumption, duration of diagnosis, perception of health status, anxiety, and depression were significant factors influencing CVD patients’ health-promoting behaviors. Based on the study findings, it is suggested that nursing intervention programs be developed that empower patients with CVD to improve health-promoting behaviors. Future research should evaluate nursing intervention studies with CVD patients that address the five subdomains of health-promoting behaviors. This study found that women are better at adhering to dietary habits and health-promoting behaviors than men. Therefore, effective health-promoting programs should be based on different needs and situations of gender.

## Figures and Tables

**Table 1:** Participants’ general characteristics and differences in health promoting behavior according to the general characteristics (*N* = 161).

				Health Promoting Behavior					
				Total	Adherence to Medication	Adherence to Dietary Habits	Adherence to Physical Activity	Adherence to Medical Recommendation	Adherence to Nonsmoking
Variable	Category	*n* (%)	Mean (*SD*)	Mean (*SD*)	Mean (*SD*)	Mean (*SD*)	Mean (*SD*)	Mean (*SD*)	Mean (*SD*)
Age	Range 29–87		66.3 (10.7)						
Gender	Male	81 (50.3)		2.59 (0.38)	2.82 (0.45)	2.54 (0.43)	2.45 (0.55)	2.60 (0.43)	3.33 (0.88)
	Female	80 (49.7)		2.74 (0.43)	2.77 (0.59)	2.79 (0.53)	2.60 (0.58)	2.74 (0.54)	3.13 (1.22)
	*t(p)*			−2.395 (0.018)	0.628 (0.531)	−3.319 (0.001)	−1.570 (0.118)	−1.819 (0.071)	1.161 (0.248)
Marital status	Married	139 (86.3)		2.65 (0.42)	2.79 (0.52)	2.67 (0.51)	2.49 (0.59)	2.64 (0.51)	3.15 (1.11)
	Single/divorced	22 (13.7)		2.74 (0.36)	2.81 (0.56)	2.62 (0.41)	2.73 (0.41)	2.81 (0.37)	3.72 (0.45)
	*t(p)*			1.038 (0.308)	0.153 (0.879)	−0.449 (0.656)	2.390 (0.022)	1.840 (0.074)	4.190 (<0.001)
Education	No education or elementary school	43 (26.7)		2.69 (0.28)	2.83 (0.62)	2.67 (0.33)	2.61 (0.54)	2.62 (0.34)	3.23 (1.28)
	Middle school	12 (7.5)		2.81 (0.27)	3.04 (0.33)	2.62 (0.34)	2.82 (0.42)	3.12 (0.44)	3.00 (1.27)
	High school	49 (30.4)		2.48 (0.35)	2.64 (0.56)	2.53 (0.48)	2.22 (0.35)	2.52 (0.57)	3.06 (1.02)
	Undergraduate/graduate school	57 (35.4)		2.77 (0.51)	2.85 (0.39))	2.78 (0.62)	2.66 (0.67)	2.74 (0.47)	3.43 (0.84)
	*F(p)*			5.470 (0.001) c < d	2.586 (0.55)	2.104 (0.102)	8.009 (<0.001) a,b,d > c	5.699 (0.001) a < b, b > c	1.321 (0.270)
Economic status	≥Middle	112 (69.6)		2.69 (0.40)	2.86 (0.050)	2.64 (0.466))	2.57 (0.580)	2.770 (0.506)	3.36 (0.96)
	Low	49 (30.4)		2.59 (0.43)	2.65 (0.068)	2.71 (0.753)	2.41 (0.550)	2.449 (0.398)	2.93 (1.23)
	*t(p)*			1.395 (0.165)	2.317 (0.022)	−0.806 (0.422)	1.695 (0.092)	3.932 (<0.001)	2.366 (0.019)
Alcohol consump-tion	No	95 (59.0)		2.74 (0.45)	2.81 (0.53)	2.77 (0.53)	2.61 (0.60)	2.78 (0.56)	3.17 (1.22)
	Yes	66 (41.0)		2.55 (0.32)	2.78 (0.50)	2.51 (0.40)	2.40 (0.51)	2.51 (0.32)	3.31 (0.80)
	*t(p)*			3.093 (0.002)	0.344 (0.731)	3.189 (0.002)	2.301 (0.023)	3.541 (0.001)	−0.812 (0.418)
Duration from diagnosis of CVD (years; *M* and *SD*)	1–10	110 (68.3)		2.61(0.42)	2.80(0.51)	2.59(0.50)	2.43 (0.59)	2.71 (0.51)	3.22 (1.04)
	≥10	51 (31.7)		2.77 (0.38)	2.78 (0.55)	2.81 (0.46)	2.72 (0.48)	2.58 (0.46)	3.25 (1.12)
	*t(p)*			−2.197 (0.029)	0.197 (0.844)	−2.566 (0.011)	−3.032 (0.003)	1.465 (0.145)	−0.152 (0.879)
Comor-bidities	0	43 (26.7)		2.62 (0.41)	2.738 (0.63)	2.69 (0.55)	2.45 (0.56)	2.52 (0.24)	3.20 (0.94)
	1	85 (52.8)		2.68 (0.39)	2.803 (0.51)	2.66 (0.43)	2.57 (0.56)	2.72 (0.52)	3.11 (1.16)
	≥2	33 (20.5)		2.68 (0.48)	2.879 (0.35)	2.65 (0.60)	2.50 (0.62)	2.72 (0.61)	3.57 (0.90)
	*F(p)*			0.262 (0.77)	1.329 (0.251)	0.064 (0.938)	0.663 (0.517)	2.687 (0.071)	2.233 (0.111)
Depression	≤15 Normal	78 (48.4)		2.76 (0.42)	2.91 (0.43)	2.76 (0.53)	2.59 (0.56)	2.80 (0.52)	3.30 (1.12)
	≥16 Depression	83 (51.6)		2.58 (0.45)	2.69 (0.57)	2.58 (0.45)	2.46 (0.58)	2.54 (0.43)	3.16 (1.02)
	*t(p)*			−2.831 (0.005)	−2.758 (0.006)	−2.300 (0.023)	−1.378 (0.170)	−3.456 (0.001)	−0.823 (0.412)

Note. a = no education or elementary school; b = middle school; c = high school; d = undergraduate/graduate school; CVD: cardiovascular disease; *SD*, standard deviation.

**Table 2:** Levels of depression, anxiety, perception of health status, and health-promoting behaviors. (*N* = 161).

Variable	Range	Mean(*SD*)
Health-promoting behavior	1.80–3.67	2.66 (0.41)
Adherence to dietary habits	1.46–3.85	2.66 (0.50)
Adherence to physical activity	1.38–3.88	2.52 (0.57)
Adherence to nonsmoking	1–5	3.23 (1.06)
Adherence to medication	1–4	2.80 (0.52)
Adherence to medical recommendation	1.75–4	2.67 (0.49)
Perception of health status	1–5	2.61 (1.01)
Anxiety	1.2–4	2.15 (0.37)
Depression	0–53	21.72 (10.00)

*SD*, standard deviation.

**Table 3:** Correlations among depression, anxiety, perception of health status, and health-promoting behavior (*N* = 161).

Variable	Depression	Anxiety	Perception of Health Status	Health-Promoting Bhavior
Depression	—			
Anxiety	0.026	—		
Perception of health status	−0.428 **	0.091	—	
Health-promoting behavior	−0.323 **	0.105	0.203 **	—

** *p* < 0.05.

**Table 4:** Factors affecting health-promoting behavior (*N* = 161).

	Health-Promoting Behavior (Total)	Adherence to Medication	Adherence to Dietary Habits	Adherence to Physical Activity	Adherence to Medical Recommendation	Adherence to Nonsmoking
Variable	β (*SE*)	β (*SE*)	β (*SE*)	β (*SE*)	β (*SE*)	β (*SE*)
Constant	2.143 (0.192)		1.725 (0.197) **	2.214 (0.216) **	3.118 (0.190) **	5.851 (0.832) **
Gender			0.198 (0.076) **			
Marital status						−0.233 (0.241) **
Economic status					−0.301 (0.076) **	−0.147 (0.171) *
Alcohol consumption	−0.204 (0.061) **			−0.174 (0.087) **	−0.251 (0.072) **	
Duration from diagnosis of CVD	0.245 (0.067) **		0.230 (0.084) **	0.248 (0.092) **		
Perception of health status	0.183 (0.030) **	0.362 (0.038) **		0.200 (0.042) **		0.293 (0.077) **
Anxiety						−0.262 (0.011) **
Depression	0.232 (0.063) **	0.165 (0.076) **	0.213 (0.078) **		0.227 (0.071) **	
*R* ^2^	0.179	0.174	0.135	0.127	0.217	0.194
Adj. *R*^2^	0.158	0.164	0.118	0.110	0.202	0.173
*F*	8.487 **	16.639 **	8.146 **	7.607 **	14.529 **	9.395 **

CVD, cardiovascular disease; SE, standard error; * *p* < 0.05. ** *p* < 0.01.

## Data Availability

The data presented in this study are available on request from the corresponding author. The data are not publicly available due to privacy.
